# Constraints on oceanic methane emissions west of Svalbard from atmospheric in situ measurements and Lagrangian transport modeling

**DOI:** 10.1002/2016JD025590

**Published:** 2016-12-10

**Authors:** I. Pisso, C. Lund Myhre, S. M. Platt, S. Eckhardt, O. Hermansen, N. Schmidbauer, J. Mienert, S. Vadakkepuliyambatta, S. Bauguitte, J. Pitt, G. Allen, K. N. Bower, S. O'Shea, M. W. Gallagher, C. J. Percival, J. Pyle, M. Cain, A. Stohl

**Affiliations:** ^1^ NILU‐Norwegian Institute for Air ResearchKjellerNorway; ^2^ Centre for Arctic Gas Hydrate, Environment and Climate, Department of GeologyUiT‐The Arctic University of NorwayTromsøNorway; ^3^ FAAMNatural Environment Research CouncilCranfieldUK; ^4^ School of Earth, Atmospheric and Environmental SciencesUniversity of ManchesterManchesterUK; ^5^ National Centre for Atmospheric ScienceUK; ^6^ Department of ChemistryUniversity of CambridgeCambridgeUK

**Keywords:** Arctic methane, emission flux, inversion, hydrates, Lagrangian transport

## Abstract

Methane stored in seabed reservoirs such as methane hydrates can reach the atmosphere in the form of bubbles or dissolved in water. Hydrates could destabilize with rising temperature further increasing greenhouse gas emissions in a warming climate. To assess the impact of oceanic emissions from the area west of Svalbard, where methane hydrates are abundant, we used measurements collected with a research aircraft (Facility for Airborne Atmospheric Measurements) and a ship (Helmer Hansen) during the Summer 2014 and for Zeppelin Observatory for the full year. We present a model‐supported analysis of the atmospheric CH_4_ mixing ratios measured by the different platforms. To address uncertainty about where CH_4_ emissions actually occur, we explored three scenarios: areas with known seeps, a hydrate stability model, and an ocean depth criterion. We then used a budget analysis and a Lagrangian particle dispersion model to compare measurements taken upwind and downwind of the potential CH_4_ emission areas. We found small differences between the CH_4_ mixing ratios measured upwind and downwind of the potential emission areas during the campaign. By taking into account measurement and sampling uncertainties and by determining the sensitivity of the measured mixing ratios to potential oceanic emissions, we provide upper limits for the CH_4_ fluxes. The CH_4_ flux during the campaign was small, with an upper limit of 2.5 nmol m^−2^ s^−1^ in the stability model scenario. The Zeppelin Observatory data for 2014 suggest CH_4_ fluxes from the Svalbard continental platform below 0.2 Tg yr^−1^. All estimates are in the lower range of values previously reported.

## Key Points

- Measurements around Svalbard show no atmospheric methane (CH_4_) enhancements from CH_4_ releases into seawater from the ocean floor
- We provide an upper limit for oceanic CH_4_ emissions compatible with the lack of an observable atmospheric signal
- Sea‐air emission fluxes of CH_4_ are small offshore Svalbard, both for transfer via bubbles and via diffusive flux of dissolved CH_4_

## 1 Introduction

Natural and anthropogenic greenhouse gases (GHGs) warm Earth's climate. Methane (CH_4_) is a major GHG, with both anthropogenic and natural emissions. Such emissions are sensitive to climate feedbacks [*Ciais et al*., 2013]. CH_4_ has a large impact on the Earth's radiative balance (its 100 year global warming potential, i.e., its radiative impact relative to CO_2_, is 28–34 [*Myhre et al*., 2013]), a strong indirect effect as a precursor to tropospheric ozone and also impacts its own lifetime [*Isaksen*, 1988; *Isaksen et al*., 2011]. CH_4_ emissions from the ocean constitute an estimated 2–14.4% of the global atmospheric CH_4_ budget [*Hovland et al*., 1991; *Lambert and Schmidt*, 1993]. A large part of the CH_4_ reservoir in the ocean is stored in the form of CH_4_ hydrates within the ocean floor. Methane hydrates (or more specifically methane clathrate hydrates) are crystalline compounds in which CH_4_ is trapped in a lattice of water ice (e.g., 4CH_4_ · 23H_2_O) [*Sloan and Koh*, 2008]. They are stable in solid form within marine sediments at low temperature and high pressure and occur at all continental margins and in permafrost regions of the Earth [*Collett et al*., 2009]. An estimated 1.55 × 10^5^ Tg of CH_4_ (total CH_4_ mass) may be stored in hydrate reservoirs in the sea bed under the shallow waters of the Arctic Ocean alone [*Kretschmer et al*., 2015]. Hydrates release CH_4_ when they melt [*Mienert et al*., 2005; *Westbrook et al*., 2009], and it has been suggested that global warming could destabilize them [*Biastoch et al*., 2011; *Ferré et al*., 2012]. Nevertheless, *Kretschmer et al*. [2015] estimated that the yearly flux from hydrates, even under extreme future warming scenarios, would have only a modest impact. CH_4_ bubbles released from the seafloor can rise through the water column in plumes called flares. Flares normally cannot reach the sea surface in deep waters, but CH_4_ can also be dissolved in the water. Dissolved CH_4_ can be oxidized by methanotrophic bacteria [*Gentz et al*., 2014; *Steinle et al*., 2015] but may also be transported to the sea surface with ocean currents, followed by air‐sea exchange across the ocean surface. *Berndt et al*. [2014] showed that CH_4_ seepage has already existed for more than 1000 years.

There are large uncertainties in estimates of seabed CH_4_ emissions to the water column in the Arctic [*Arctic Monitoring and Assessment Programme (AMAP)*, 2015], with the fraction of these emissions ultimately reaching the atmosphere being even more uncertain. Emissions of CH_4_ from the seafloor off the west coast of Svalbard have been observed [e.g., *Gentz et al*., 2014; *Graves et al*., 2015], and measurement campaigns that have targeted this area have found a large number of gas flares in the water column [e.g., *Westbrook et al*., 2009; *Sahling et al*., 2014]. *Graves et al*. [2015], *Marín‐Moreno et al*. [2015], *Sahling et al*. [2014], and *Stranne et al*. [2016] have put the present and future flux into perspective. Gas hydrates might be present below the present day upper limit of gas hydrate stability around ~380 m [*Westbrook et al*., 2009], and these might be subject to dissociation due to warming. However, although there is evidence of hydrate dissociation due to glacial retreat [*Portnov et al*., 2016] in the past on the shelf at depth < 250 m the methane seeps are linked to “natural” emissions, such as deep faults [*Damm et al*., 2005; *Knies et al*., 2004; *Sahling et al*., 2014]. We carried out an interdisciplinary ocean‐atmosphere campaign to quantify CH_4_ fluxes in this area [*Myhre et al*., 2016]. Data on CH_4_ concentrations were obtained for the seafloor, within the water column, and in the atmosphere during summer 2014 [*Bünz*, 2014; *Mienert et al*., 2014; *Myhre et al*., 2016]. Using these data, *Silyakova et al*. [2015] reported a large number of gas flares in the ocean and a rich abundance of CH_4_ in the bottom waters. However, the CH_4_ concentrations in the water near the ocean surface were very low [*Silyakova et al*., 2015]. Furthermore, *Myhre et al*. [2016] estimated that the fluxes to the atmosphere from dissolved CH_4_ were 0.04 nmol m^−2^ s^−1^ (*σ* = 0.13), 4 orders of magnitude less than previous estimates for the Laptev Sea by *Shakhova et al*. [2014]. These lower estimates are consistent with the more recent estimates of 4 nmol m^−2^ s^−1^ by *Berchet et al*. [2016] and 2.2 nmol m^−2^ s^−1^ in *Thornton et al*. [2016]. In this paper, we present a model‐supported analysis of the atmospheric CH_4_ measurements during summer 2014, as part of the MOCA (methane from the ocean to the atmosphere [*Myhre et al*., 2016]), campaign as well as of data for the whole year from the Zeppelin Observatory, to constrain ocean‐atmosphere fluxes of CH_4_. In addition, we present updated estimates of the uncertainty. In the next section we describe the measurement data, the model, and the methodology used; section 3 presents the results, section 4 discusses the results in the light of other published flux estimates, and provides conclusions.

## 2 Data and Methods

### 2.1 Measurements

#### 2.1.1 Aircraft Measurements

**Figure 1:**
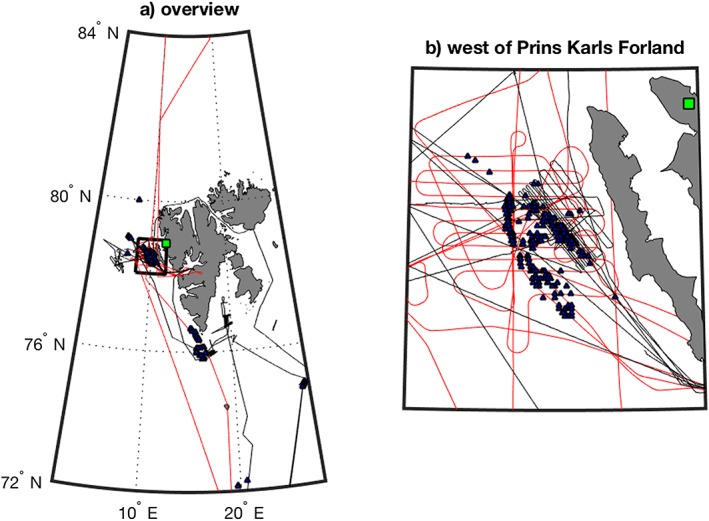
Map of the campaign area around Svalbard with the measurement locations. (a) Overview of the Svalbard archipelago and (b) zoom‐in on the area west of Prins Karls Forland where most of the intensive measurements were taken. Known gas flares in the ocean are marked with blue dots [*Sahling et al*., 2014; *Portnov et al*., 2016]. The aircraft track is shown in red, the ship track in black, and the location of the Zeppelin Observatory is marked with a green square.

Airborne measurements were performed over the Norwegian Sea using the UK FAAM (Facility for Airborne Atmospheric Measurements) BAe 146 aircraft (Figure 1a). The aircraft flew from Kiruna, Northern Sweden, to Svalbard on 1 July and back to Kiruna on 3 July 2014. We concentrate our analysis on a dedicated measurement flight at low altitudes from the airport in Longyearbyen toward the west on 2 July 2014. The aircraft flew a low‐level grid pattern over the prolific CH_4_ seepage zone at 30 m above sea level (m asl), down to 15 m asl during profiling, to record upwind and downwind differences in CH_4_ mixing ratios resulting from CH_4_ emissions (Figure 1b). CH_4_ dry mole fractions were recorded at 1 Hz with a Fast Greenhouse Gas Analyzer (Los Gatos Research). Details on how this instrument was operated on the FAAM aircraft, including the calibration and data processing procedures employed, are given by *O'Shea et al*. [2013]. The aircraft also carried a suite of instruments for measuring the concentrations of several other atmospheric trace constituents (e.g., carbon monoxide and CO). These data showed that the air masses observed during this flight were quite homogeneous, without pronounced influence from long‐range pollution transport.

#### 2.1.2 Ship‐Borne Measurements

The research vessel Helmer Hansen [*Bünz*, 2014] departed from the port of Tromsø on 20 June 2014 and returned to it on 29 July 2014. The ship was equipped to measure CH_4_ in the atmosphere (Picarro Cavity Ring Down Spectrometer (CRDS), model G2401) [*Myhre et al*., 2016]. The data are quality assured, and exhaust influenced peaks were removed using CO_2_ as a tracer. Intensive surveys took place west of Prins Karls Forland (Figure 1b). On 2 July 2014, the ship activities were coordinated with the aircraft, allowing measurement comparisons when the aircraft passed the ship at close distance. Isotopic composition of CH_4_ was also determined in air samples taken on board the ship but did not show clear evidence for emissions from hydrates [*Myhre et al*., 2016].

#### 2.1.3 Station Measurements

**Figure 2:**
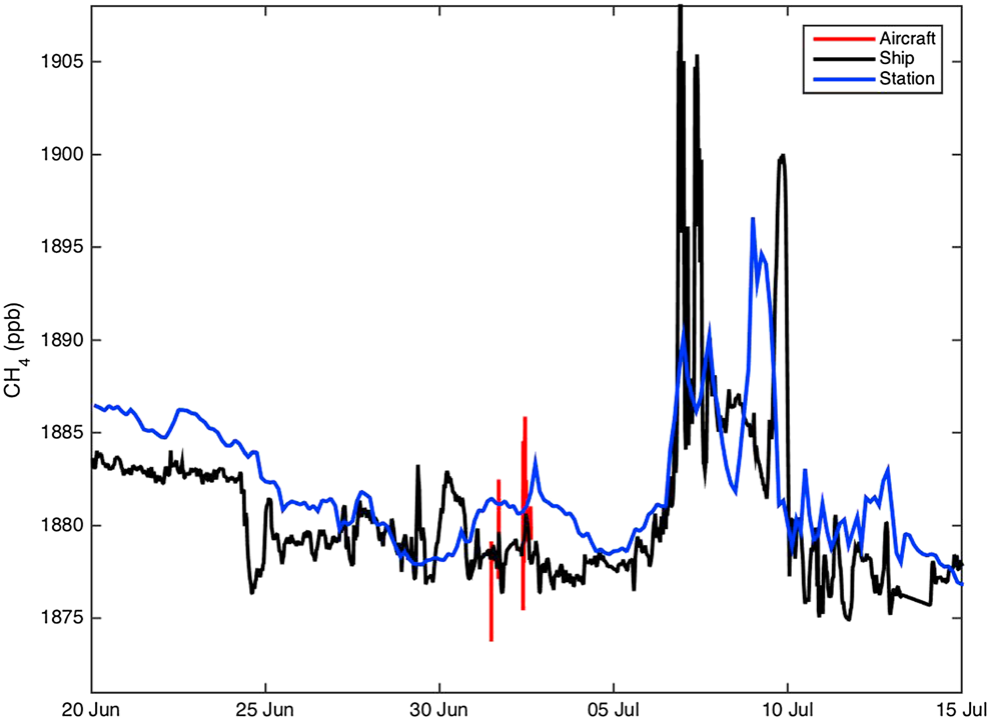
Time series of CH_4_ measurements taken at the different measurement platforms used in this study during the campaign period 20 June to 15 July 2014. The aircraft measurements (red line) were made below 100 m during flights from Longyearbyen. The ship data are shown with a black line and data from the Zeppelin Observatory with a blue line.

We also used CH_4_ data from the Zeppelin Observatory for the full year of 2014. The station is located in a largely unperturbed Arctic environment on a ridge of Zeppelin mountain on the western coast of Spitsbergen (78.91°N, 11.89°E, altitude 476 m asl, Figure 1). Contamination from the nearby small settlement of Ny Ålesund (located near sea level) can generally be neglected at Zeppelin, although pollution peaks (black carbon, particle number, ozone (decrease), and SO_2_) from cruise ships are sometimes evident in the aerosol measurements [*Eckhardt et al*., 2013]. The CH_4_ instrument on the Zeppelin Observatory is a Picarro G2401, the same instrument type as used on the ship. The inlet is atop a 15 m mast 491 m asl. Figure 2 shows time series of CH_4_ mixing ratios measured at Zeppelin, as well as ship‐borne and airborne measurements from 20 June to 15 July 2014. It can be seen that the station and ship‐borne measurements agree relatively well with each other, despite the fact that the ship was operating in a large region and, most of the time, was hundreds of kilometers away from the station. This suggests that a large fraction of the measured CH_4_ variability is caused by large‐scale processes, especially the advection of CH_4_‐rich plumes from the continent (see section 3), in agreement with studies using CH_4_ isotope data [*Fisher et al*., 2011]. For example, strong CH_4_ enhancements on 9 July 2014 detected at Zeppelin were correlated with increases in CO (an anthropogenic pollution tracer) and were caused by the transport of pollution from Europe.

### 2.2 Transport Model Calculations

To link potential emission sources and observations, we used the Lagrangian particle dispersion model FLEXPART version 9.2 [*Stohl et al.*, 1998, 2005]. The model was driven with operational meteorological analysis data from the European Centre for Medium‐Range Weather Forecasts with 0.5° × 0.5° latitude/longitude resolution globally and 0.1° × 0.1° resolution in a nest covering the area 72–84°N and 2–28°E. The resolution of the nest corresponds approximately to the spectral resolution of the European Centre for Medium‐Range Weather Forecasts model operational in 2014 (CY40R1). The model has 137 levels with 3 h resolution with 6 h analysis and 3 h forecast time steps. FLEXPART simulations 20 days backward in time were made. For the ship, small receptor “boxes” located along the ship track at 30 min intervals were generated; for the aircraft, boxes were generated whenever it changed altitude by 100 m or surface position by 0.1° latitude or longitude; for the Zeppelin Observatory, FLEXPART calculations were made at fixed 3 h intervals.

**Figure 3:**
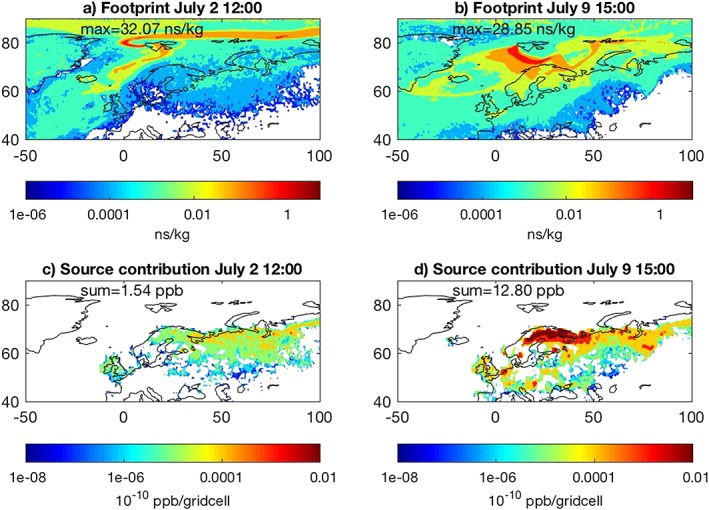
(a and b) Footprint emission sensitivities from 20 day FLEXPART backward calculations for measurements taken at the Zeppelin Observatory on 2 July 2014 at 03:00 UTC and on 9 July 2014 at 12:00 UTC. (c and d) Corresponding source contributions using an inventory of terrestrial CH_4_ sources. Total mixing ratio enhancements were 1.54 and 12.80 ppb, respectively.

The output of FLEXPART in backward mode is a gridded emission sensitivity field. Of particular interest is the emission sensitivity in the lowest model layer (the so‐called footprint layer, here below 100 m asl) as most emissions occur at the surface. The footprint emission sensitivity (e.g., in units of kg^−1^ m^2^ s for emission densities given in kg m^−2^ s^−1^) gives the sensitivity of a simulated mixing ratio at the receptor location to the emission area flux density in the source grid cell [*Seibert and Frank*, 2004]. As an example, Figures 3a and 3b show the footprint emission sensitivities for the Zeppelin Observatory calculated for 2 July and 9 July 2014. In the first case, air arrived primarily from the Arctic Ocean, whereas in the second case, the air traveled over northern Fennoscandia. The product of the emission sensitivity and a known emission flux gives source contributions, i.e., a map of the contributions to the mixing ratio at the receptor per grid cell. Figures 3c and 3d show the source contributions from terrestrial CH_4_ emissions corresponding to the 2 and 9 July footprints. Our CH_4_ land emission inventory is based on three source categories: wetlands, anthropogenic, and biomass burning emissions. The wetlands emission inventory (LPX‐Bern v1.2) is from 2014 [*Stocker et al*., 2014]. The anthropogenic emission inventory, ECLIPSE‐GAINS, is from 2010 [*Stohl et al*., 2015]. The biomass burning emission inventory is the Global Fire Emissions Database, Version 4, (GFEDv4) [*Randerson et al*., 2015]. In all three cases the inventories report the fluxes monthly. As can be seen Figures 3c and 3d, there were strong source contributions from Fennoscandia on 9 July but not on 2 July. Consequently, total CH_4_ mixing ratio enhancements at the receptor due to emissions during the past 20 days were 1.5 and 12.7 ppb for the cases of 2 and 9 July 2014, respectively.

### 2.3 Ocean Emission Scenarios

**Figure 4:**
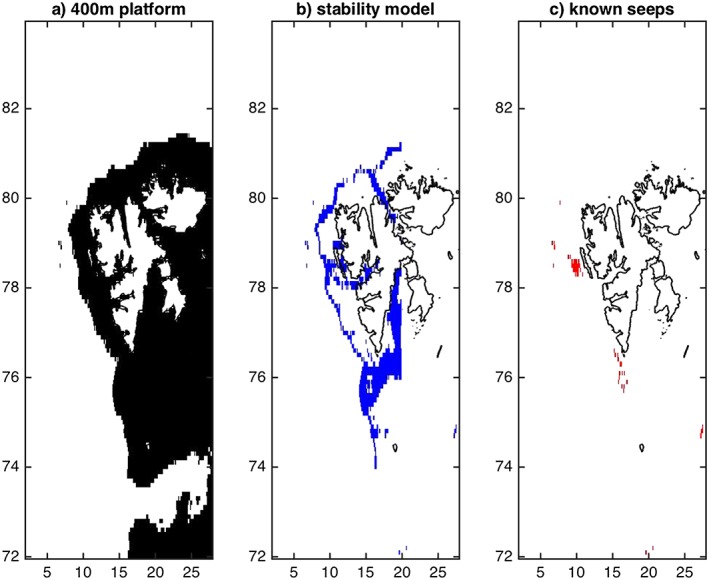
Three scenarios for potential CH4 emission regions: (a) all areas with sea depth less than 400 m; (b) modeled potentially unstable CH4 hydrates at the sea bottom; (c) known seep locations. Known seeps are also included in scenarios Figures 4a and 4b.

The aim of this study is to constrain the magnitude of the CH_4_ fluxes from the ocean areas around Svalbard where CH_4_ hydrates are found. These fluxes include, but are not restricted to, CH_4_ originating form hydrate decomposition. Estimation of CH_4_ hydrate fluxes requires knowledge of areas of hydrate instability. Furthermore, it must be assumed that emissions to the atmosphere occur in the region where the CH_4_ hydrate decomposition occurs, whether directly by bubble transfer of CH_4_ to the surface [*Veloso et al*., 2015] or via vertical flux of dissolved CH_4_ in the ocean and subsequent gas transfer to the atmosphere. Unfortunately, the available information on the distribution of CH_4_ sources in the seabed is incomplete, and knowledge of where exactly CH_4_ hydrates decompose is even more limited. Therefore, we developed three scenarios to delineate the potential source regions where such emissions may occur. For all three scenarios, we first defined an area around Svalbard from 2–28°E to 72–84°N where we had enough atmospheric measurement data to constrain the fluxes (Figure 4).

In the first and most simple scenario, we used a bathymetry map of the ocean to identify areas with water depth less than 400 m. This is based on the finding that CH_4_ seabed sources exist around Svalbard [e.g., *Westbrook et al*., 2009; *Sahling et al*., 2014] and is intended as an extreme scenario for uncertainty assessment purposes assuming that transfer of CH_4_ to the surface cannot occur at deeper zones (Figure 4a). The second scenario (Figure 4b) is based on results from the CH_4_ hydrate stability model CSMHYD (Colorado School of Mines HYDrates [*Sloan and Koh*, 2008]) which estimates whether hydrate is stable at a particular location, taking into account the ocean bottom temperatures (World Ocean Database, 2013, https://www.nodc.noaa.gov/OC5/WOD13), thermal gradients (Global Heatflow Database) and their uncertainties (±2 °C in ocean bottom temperatures and ±10 °C km^−1^ in thermal gradients). Locations where the hydrate stability zone outcrops at the seabed are considered potential CH_4_ seep locations (Figure 4b). The third scenario (Figure 4c) is based on the location of known seeps where bubbling of CH_4_ from the seafloor has been documented previously [*Lammers et al*., 1995; *Westbrook et al*., 2009; *Sahling et al*., 2014; *Panieri et al*., 2015; *Portnov et al*., 2016], gridded to a resolution of 0.1° × 0.1°, which was also used for the other two scenarios. The known seep locations—if not already included—were also added to scenarios 1 and 2. The areas of the three scenarios are 228,484 km^2^, 28,780 km^2^, and 1644 km^2^, respectively. In section 3, we use measurement data to constrain emission fluxes for the three areas.

### 2.4 Inverse Modeling of Emission Fluxes and Background Concentrations

The measured CH_4_ mixing ratios can be modeled given the emission fluxes, the source sensitivity (the footprints), and the background concentrations at the measurement locations. In matrix form:

$$y_{m}=Mx_{f}+y_{bkg}\text{,}$$

where **y**_m_is the modeled mixing ratio at the measurement location (ppb), **M** is the source‐receptor relationship from FLEXPART source sensitivities (ppb nmol^−1^ m^2^ s), **x**_f_ is the emission flux (nmol m^−2^ s^−1^), and **y**_bkg_ are the background mixing ratios resulting from emissions and sink processes occurring prior to the 20 days of FLEXPART backward simulations (ppb). Notice that **Mx**_f_ corresponds to the source contributions described in section 2.2.

The same can be expressed as

$$y_{m}=Hx_{a}$$

defining the augmented state vector $x_{a}=\left(\begin{array}{c} x_{f}\\ y_{bkg} \end{array}\right)$ and **H** = (**M**, 1).

Without prior knowledge of the sources and background, the best least squares estimate of **x**_a_ from the observations **y**_o_ is given by **H** ^+^ **y**_o_ where **H** ^+^ is the Moore‐Penrose pseudo inverse of **H**.

## 3 Results

### 3.1 Case Study: Aircraft Based CH_4_ Flux Estimates

**Figure 5:**
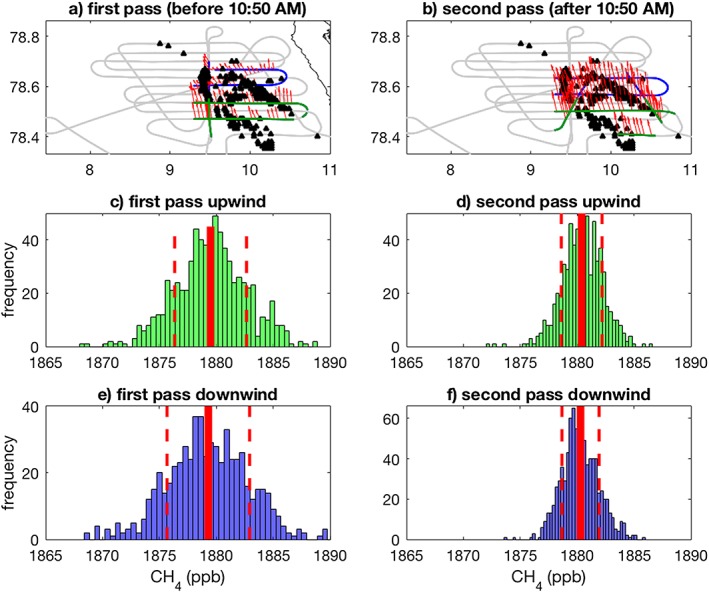
The upwind and downwind measurement locations for the (a) first and (b) second parts of the flight on 2 July 2014. The flight track is shown in gray. A superimposed green (blue) line indicates upwind (downwind) CH_4_ measurements. Black triangles mark the locations of known seeps. Frequency distributions of 1 Hz CH_4_ measurements taken (c and d) upwind (green) and (e and f) downwind (blue) of the known seep locations for the first part (Figures 5c and 5e) and second part (Figures 5d and 5f) of the flight on 2 July 2014. The red vertical bars represent the mean values. Dashed red lines represent 1 sigma standard deviations.

We first present a case study based on measurement data taken during the BAe 146 flight on 2 July 2014 [*Myhre et al*., 2016]. To avoid the potential impact of slightly changing CH_4_ mixing ratios in the inflowing air masses during the duration of the flight, we analyzed the two halves of the flight separately (Figures 5a and 5b).

In order to perform an upwind vs downwind measurements comparison, we selected the measurements below 1000 m for both passages over the known seeps region. Each subset consists of four flight segments aligned east west. In both cases we use the two northernmost legs as downwind samples and the two southernmost legs as upwind samples, based on the prevailing southerly wind direction. Figures 5c–5f show a comparison between the frequency distributions of upwind and downwind measurements for both halves of the flight. The upwind and downwind mixing ratios are only marginally different (and well within both the 1 standard deviation variability of the sampled data), with differences of −0.27 ppb and 0.16 ppb for the first and second parts of the flight, respectively. Given the variability in the data, in both cases the differences between upwind and downwind data subsets are not statistically significant according to a Welch's unequal variances (unpaired) *t* test (see Table S1 in the supporting information). The data thus suggest that the seep areas did not have a detectable influence on the atmospheric CH_4_ mixing ratios. Nevertheless, we perform a source strength estimation based on the upwind/downwind mixing ratios to provide am upper boundary. Using these upwind/downwind differences, we can estimate the source strength by a simple budget calculation (equation (1)) [*Karion et al*., 2013]:

(1) $$F=\frac{\Delta C\times H\times v\times \cos\left(\theta \right)}{l}$$

Here *F* is the CH_4_ flux in the seep area, Δ*C* is the upwind/downwind concentration difference, *H* is the mixing height, *v* the mean measured wind speed component in the upwind‐downwind direction, *θ* is the angle between the aircraft track and the direction of the wind, and *l* is the average distance between upwind and downwind measurements. The mixing height *H* was estimated from aircraft vertical profiles of CH_4_ to be ~1000 m (see Figure S1 in the supporting information), the average wind speed was 4 m s^−1^ for both halves of the flight, *l* is 40 km, and the wind angles are ~130°. This gives estimates of F = −0.1 nmol m^−2^ s^−1^ and 0.02 nmol m^−2^ s^−1^ for the first and second parts of the flight, respectively.

In order to estimate the uncertainty of the case study flux estimates, we combined the standard error of the mean for the upwind and downwind measurements for both flight halves (0.37 ppb and 0.15 ppb, respectively) and the maximum estimated instrument drift (1.41 ppb). Using the uncertainties in the other parameters (Δ*l* = 10 km, Δ*h* = 100 m, Δ*v* = 1 m^−2^ s^−1^, Δ*θ* = 15*°*) and the standard error of the mean of the data, we obtain ranges of uncertainty of −0.9 to 0.6 nmol m^−2^ s^−1^ for the first part and −0.1 to 1.3 nmol m^−2^ s^−1^ for the second part of the flight. The full range of uncertainty including all parameters and the maximum possible drift is −7.6 to 9.2 nmol m^−2^ s^−1^ for the first part and −3.7 to 9.7 for the second part of the flight. This is a conservative estimate considering the worst‐case scenario as described in the supporting information (Text S1).

### 3.2 Transport Model Analysis

The upwind versus downwind comparison presented in the previous section is based solely on measured values and involves several simplifying assumptions. Furthermore, we could only explore the emissions in a very small region scanned by the aircraft during a very short period. To take advantage of the full data set to explore emissions surrounding Svalbard during the whole year 2014, we use the emission sensitivities calculated by FLEXPART to constrain the emission fluxes.

#### 3.2.1 Modeled Atmospheric CH_4_ Enhancements From Oceanic and Land Sources

**Figure 6:**
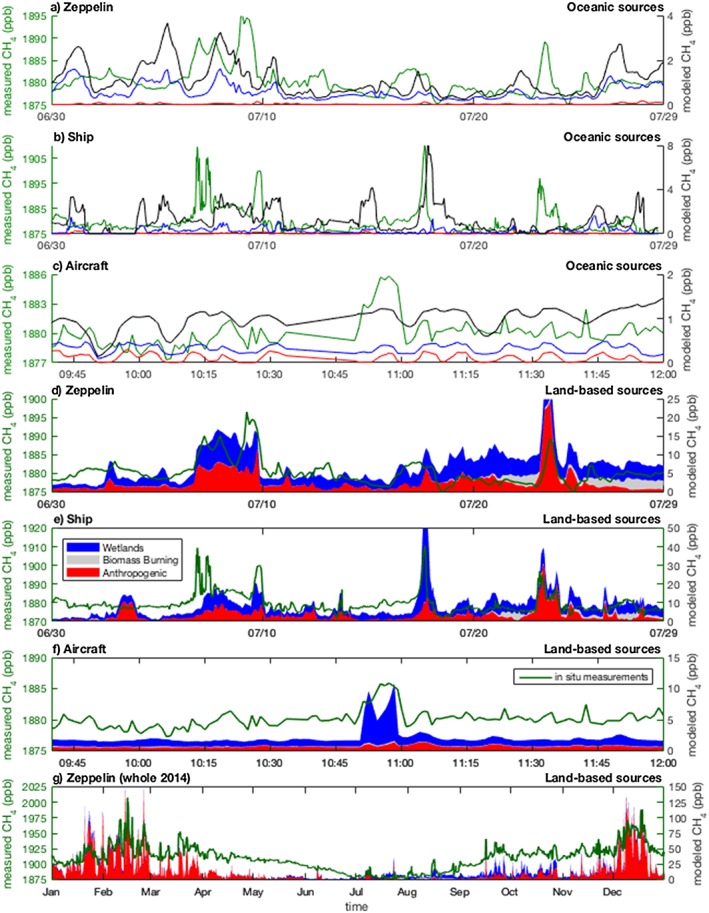
Time series of measured CH_4_ mixing ratios (green line, left axis scale) and modeled CH_4_ mixing ratio enhancements with respect to the background concentration (right axis scale) simulated with FLEXPART. In the first three panels the simulations assume a constant CH_4_ flux density of 1 nmol m^−2^ s^−1^ in each of the three potential oceanic emission source regions (see Figure 3), for (a) the Zeppelin Observatory, (b) the ship campaign, and (c) the research flight on 2 July 2014. The line colors correspond to the emission scenarios shown in Figure 3, black for sea depth smaller than 400 m, blue for the CH_4_ hydrate stability model, and red for the known seeps. CH_4_ enhancements simulated with FLEXPART and terrestrial CH_4_ emissions from anthropogenic sources (red), fires (gray), and wetlands (blue) for (d) Zeppelin (July 2014 only), (e) Helmer Hansen research vessel, (f) FAAM aircraft, and (g) Zeppelin during the full year 2014.

We calculated the footprint emission sensitivities for the whole measurement data set with FLEXPART as described in section 2.2. Multiplying these sensitivities with the emissions from the three scenarios of section 2 (assuming a constant flux of 1 nmol m^−2^ s^−1^), we modeled CH_4_ enhancements per nmol m^−2^ s^−1^ flux for the 20 day period of the backward calculations, for each of the three scenarios. This allows us to identify periods when the sampled air was potentially influenced by oceanic emissions in the three regions. Figures 6a–6c show the scenario‐based model results for the three measurement platforms. The correlation coefficients of the modeled oceanic CH_4_ contributions (for the three different emission scenarios) with the measured CH_4_ (all data platforms considered together) are only 0.07, 0.13, and 0.1 for the known seeps, the stability model, and the 400 m sea depth scenario, respectively (see Table S2). This suggests that the oceanic emissions in our three emission regions are too small to produce a clear signal in the measurements. In fact, Figures 6d and 6e show CH_4_ mixing ratios calculated using FLEXPART and terrestrial CH_4_ emissions from three different land source types (anthropogenic, biomass burning, and wetlands), and these show higher correlation with the measurements (0.32, −0.16, and 0.42, respectively), suggesting that terrestrial sources mainly cause the measured variability as mentioned in section 2.2 and illustrated in Figure 3. At Zeppelin (Figure 6d), there were a few episodes with enhanced measured CH_4_ mixing ratios and all of them appear to be related to long‐range transport from continental sources as mentioned earlier and shown in Figures 2 and 3. The highest peaks in the Helmer Hansen measurements (Figure 6e) are associated with transport from continental sources as well. The flight on 2 July 2014 was not much (<2 ppb most of the time) influenced by continental sources (Figure 6f).

Figure 6g shows a comparison of the measured CH_4_ mixing ratios for the Zeppelin Observatory for the whole year 2014 with modeled enhancements due to terrestrial emissions. The measured CH_4_ shows a strong seasonality in the background, which is mainly due to seasonal variations in atmospheric OH concentrations. Superimposed on this background, there are numerous episodes with strong enhancements. As the comparison with the FLEXPART results shows, most of these enhancements can be well explained with transport of terrestrial emissions. In winter, these are predominantly anthropogenic, while in late summer/autumn both anthropogenic and wetland emissions contribute. The correlation (*r* = 0.71) between the measurements and the model is higher for the land contributions than for the oceanic contributions, even though the model does not account for the seasonally varying CH_4_ background. This is in agreement with *Berchet et al*. [2016] although for a different region, a different year, using a different transport model, different landmass emissions sources, and a different representation of the hemispheric baseline.

#### 3.2.2 Upwind and Downwind Concentrations From Transport Modeling

To obtain an upper constraint on the oceanic emissions in the absence of a clear correlation between the measurements and modeled tracers, we use a method that extends that of the case study in section 3.1. We select the measured CH_4_ mixing ratios that are associated with the lowest 20% and the highest 20% of the emission sensitivity values in the potential emission sensitivity regions. Analogous to the case study in section 3.1, we consider the former data set as the upwind data (i.e., data minimally influenced by emissions in CH_4_ seep areas) and the latter data set as the downwind data (i.e., data most strongly influenced by potential CH_4_ seep emissions).

In contrast to the case study of section 3.1, an unpaired *t* test shows that the mean measured mixing ratios of the downwind and upwind data set are significantly different (see supporting information, Tables S3 and S4). We calculate the uncertainty ranges by subtracting the standard error from the upwind data and adding the standard error to the downwind data set. Combining the data of the three platforms between 20 June 2014 and 31 July 2014, we find that the downwind data may be increased by at most 3.31, 1.70, and 1.32 ppb, for the three emission scenarios (see Table 1).

**Table 1:** CH_4_ Flux Constraints for the Three Different Scenarios and Data Subsets^a^

Scenario	Known Seeps				Stability Model				400 m Sea Depth			
Estimate (20% Most Sensitive)	Average Sensitivity (ppb nmol^−1^ m^−2^ s^−1^)	CH_4_ Increase Downwind (ppb)	Flux Constraint		Average Sensitivity (ppb nmol^−1^ m^2^ s)	CH_4_ Increase Downwind (ppb)	Flux Constraint		Average Sensitivity (ppb nmol^−1^m^2^ s)	CH_4_ Increase Downwind (ppb)	Flux Constraint	
			Flux Density (nmol m^−2^ s^−1^)	Total Emission Gg/yr			Flux Density (nmol m^−2^ s^−1^)	Total Emission Gg/yr			Flux Density (nmol m^−2^ s^−1^)	Total Emission Gg/yr
All platforms	0.18	3.31	18.32	15.32	0.68	1.70	2.50	36.66	1.32	1.32	0.54	62.89
Plane	0.21	1.96	9.21	7.70	0.39	3.58	9.21	134.86	2.96	2.96	1.56	181.46
Ship	0.19	4.02	21.50	17.98	0.62	1.34	2.18	31.88	2.58	1.06	0.41	47.88
Zeppelin	0.07	1.19	18.15	15.17	0.85	1.14	1.34	19.58	1.79	2.47	1.38	160.77
All platforms (land sources subtracted)	0.17	4.34	26.18	21.89	0.70	4.27	6.08	89.04	2.89	−0.97	−0.33	−38.90

a The average sensitivity is defined as the difference of the sensitivity means (in ppb nmol^−1^ m^2^ s) for the 20% most and least sensitive points. The last row is analogous to the first but with modeled contribution from terrestrial emission sources subtracted from the measurements.

#### 3.2.3 Estimates for CH_4_ Emissions into the Atmosphere During 2014

Instead of the simple box model (equation (1)) used in section 3.1, we can now use the FLEXPART mean emission sensitivity difference for the upwind and downwind data. Using this difference, we convert the maximum possible mixing ratio enhancements into maximum possible CH_4_ fluxes in the emission regions. We find that the downwind mixing ratio increases correspond to a flux of 2.50 nmol m^−2^ s^−1^ using the stability model between 20 June 2014 and 31 July 2014. The other two emission regions (known seeps and 400 m sea depth) provide a range of values from 0.54 to 18.32 nmol m^−2^ s^−1^. The different scenarios allow assessment of the uncertainty arising from the lack of knowledge in the distribution of the sources. The scenario with the largest area provides a smaller flux density, but due to the spatial inhomogeneity of the footprints (Figure 3), the relationship between the area and the flux density estimate is not linear. Performing the same analysis for three data subsets (aircraft, ship, and Zeppelin) separately, we find slightly different results (see Table 1). The strongest constraint is provided by the plane data set (0.21 ppb nmol^−1^ m^2^s). An exception arises in the stability model scenario, where Zeppelin data provide the strongest constraint due to the station's central location in the hydrate stability source region.

In order to obtain a yearly flux estimate, instead of restricting the data set to the campaign period we use Zeppelin data all throughout 2014. For every month, an upwind versus downwind analysis was performed. The sum of the individual months yields the total flux for the full year. The total CH_4_ flux obtained for the stability model scenario is of the order of 0.2 Tg yr^−1^ with a range of uncertainty between 0 and 1 Tg yr^−1^ given by the other emission scenarios. See Table S5 in the supporting information for further details.

In addition to the analysis performed so far and in order to assess the influence of the background, we can correct for the compounding influence of terrestrial CH_4_ sources. We modeled the contribution from terrestrial emissions (Figures 5d–5g) and subtracted it from the measured CH_4_ mixing ratios. After estimating the fluxes using upwind versus downwind concentrations as described above, the analysis yields similar results. See Table S6 in the supporting information for further details.

### 3.3 Estimates From Elementary Inverse Modeling

As an alternative to the previous analysis and in order to verify that the results are robust, the measurements **y**_o_ can be used to constrain CH_4_ emission fluxes and background $x_{a}=\left(\begin{array}{c} x_{f}\\ y_{bkg} \end{array}\right)$, using a pseudo inverse optimization of fluxes and background, and the measurements from the aircraft, ship, and the Zeppelin Observatory (see method description in section 2.4). No prior information is assumed. For a robust estimate and because of the lack of information on space or time variations of the source, we invert the source‐receptor relationship assuming a temporally constant flux in the three regions of interest. We used ***n*** = 2260 measurements from 20 May 2014 to 2 August 2014. The dimensions of the vectors are such that **y**_o_ has *n* components and **x** _**f**_ and **y**_bkg_ are scalar values (in symbols **y**_o_ ∈ ℝ^***n*** × 1^ in ppb, **x**_**f**_ ∈ ℝ in nmol m^−2^ s^−1^, **y**_bkg_ ∈ ℝ in ppb, and **x**_a_ ∈ ℝ^2 × 1^). The three different scenarios yield three different time series of footprints that can be interpreted as three different transport operators or source‐receptor relationships (SRRs) **M**_**s**_ ∈ ℝ^1 × ***n***^. For each emission scenario SRR there is a corresponding augmented operator **H**_s_ ∈ ℝ^2 × ***n***^ that yields a corresponding overdetermined linear problem $\left(\begin{array}{c} x_{f}\\ y_{bkg} \end{array}\right)=H_{s}^{+}y_{o}$. The solutions for the mean flux **x**_**f**_ and the background **y**_bkg_ are 1.29 nmol m^−2^ s^−1^ and 1879.50 ppb for the stability model scenario. The range of uncertainty is given by results obtained for the 400 m sea depth scenario (0.5 nmol m^−2^ s^−1^ and 1879.34 ppb) and for the known seeps scenario (9.91 nmol m^−2^ s^−1^ and 1879.37 ppb). This analysis complements the results from section 3.2.3 using the full set of measurements. In contrast, the analysis in section 3.2.3 uses only 40% of the measurements (the 20% most and least sensitive). The order of magnitude of the estimated fluxes for the three scenarios is consistent for both methods even though the second method also optimizes the value of the average background CH_4_ mixing ratio.

## 4 Summary and Discussion

In this paper, we estimated CH_4_ emission fluxes from the ocean around Svalbard using different aircraft, ship, and station data, for different definitions of possible CH_4_ seep areas, and using different methods. Using only aircraft data from a dedicated measurement flight, we find that the CH_4_ fluxes in an area of known active seeps ranged between −0.1 and 0.02 nmol m^−2^ s^−1^. We estimate the range of uncertainty of these fluxes between −0.9 nmol m^−2^ s^−1^ and 1.3 nmol m^−2^ s^−1^ based on the standard error of the mean. Taking into account the maximum possible drift of the instrument, the uncertainty ranges between −7.6 nmol m^−2^ s^−1^ and 9.7 nmol m^−2^ s^−1^.

Combining the transport model with all the data from the aircraft, ship, and the Zeppelin Observatory collected between 20 June 2014 and 31 July 2014, we obtained a CH_4_ flux estimate of 2.50 nmol m^−2^ s^−1^ for the stability model scenario. The uncertainty range of this estimate is 0.54 to 18.32 nmol m^−2^ s^−1^. Inverse modeling results for the same period yield an estimate of 1.3 nmol m^−2^ s^−1^ with a range of uncertainty between 0.5 and 9.91 nmol m^−2^ s^−1^ using the other two emission regions.

Our case study estimates based on standard error can be compared to the results presented by *Myhre et al*. [2016], who estimate the uncertainty by standard deviation. They reported a maximum flux of 14.1 nmol m^−2^ s^−1^ for 2 July 2014: larger than the value calculated in this work. For the stability model scenario, *Myhre et al*. [2016] already placed an upper limit of 2.4–3.8 nmol m^−2^ s^−1^, which is comparable to our maximum value, obtained with a more comprehensive analysis. *Myhre et al*. [2016] presented a synoptic overview of the Methane emissions from the arctic ocean to the atmosphere: present and future climate effects (MOCA) 2014 campaign. This included a lot of oceanic data. On the other hand, only a subset of the atmospheric data was used (e.g., only data from the core period of the campaign were used) and the purpose of the Lagrangian modeling was restricted to providing one single averaged sensitivity for all regions and measurement platforms. No comparison between model results and measurement data was shown, and land‐based sources were not analyzed. In this paper we presented a much more complete analysis of the modeling and data. We added data for the whole year of 2014, used alternative (and more comprehensive) methods for estimating the fluxes, compared the model results and observations, and included also an analysis of the contribution of land‐based sources. We have shown that the land‐based sources dominate the CH_4_ variability even during periods with relatively little influence from such sources.

*Graves et al*. [2015] used an ocean‐atmosphere gas exchange function method [*Wanninkhof et al*., 2009] and reported methane fluxes ranging between 0.0463 and 0.2315 nmol m^−2^ s^−1^ west of Svalbard using data from July 2011 and July and August 2012. These values are lower than our inverse modelling results but consistent with our upper limits. However, while the estimate of *Graves et al*. [2015] only considers air‐sea exchange via diffusion of dissolved CH_4_, our method also includes the contribution of bubbles of gas reaching the surface. Furthermore, our estimates are representative of a much larger area than the gas exchange method, which can only constrain the fluxes along the ship track.

We can compare our upper flux estimates for Svalbard with CH_4_ fluxes of 8.47 nmol m^−2^ s^−1^ reported by *Shakhova et al*. [2010] for the East Siberian Arctic Shelf (ESAS). In another estimate, they even obtained fluxes of up to 453 nmol m^−2^ s^−1^ for the ESAS [*Shakhova et al*., 2014]. These results were obtained for a different region, with higher wind speeds than in our case study, and were possibly influenced by subsea permafrost CH_4_ emissions. Other, more recent studies of the ESAS region reported much lower fluxes of 2.2 nmol m^−2^ s^−1^ [*Thornton et al*., 2016] and 4 nmol m^−2^ s^−1^ [*Berchet et al*., 2016]. The consistent finding of low CH_4_ fluxes for both the Svalbard region [*Graves et al*., 2015; this study] and the ESAS [*Thornton et al*., 2016; *Berchet et al*., 2016] and considering especially that all these studies used totally different data and applied very different methods calls the high fluxes of *Shakhova et al*. [2010] and particularly their extrapolation to high wind speeds [*Shakhova et al*., 2010] into question.

For determining upper limits of the total emission fluxes, we multiplied our mass flux densities with the areas of the corresponding emission scenarios. Using Zeppelin data and FLEXPART for every month of the year 2014, the total CH_4_ flux estimate for the stability model scenario is 0.2 Tg yr^−1^ for the whole year. The range of uncertainty for the yearly total CH_4_ flux estimate ranges between 0 and 1 Tg yr^−1^ based on the other emission scenarios. By extrapolating from global CH_4_ emission estimates for continental shelf areas, it was estimated that Arctic shelf regions emit 1–12 Tg yr^−1^ into the atmosphere [*McGuire et al*., 2009; *AMAP*, 2015]. On the other hand, *Shakhova et al*. [2014] provided an estimate of 17 Tg yr^−1^ based on bubble size and CH_4_ content of bubble plumes measured in the East Siberian Arctic Shelf [*AMAP*, 2015]. Thus, even when using the emission area of our quite extreme 400 m depth emission scenario, our maximum possible fluxes are only a small fraction of the fluxes reported by *Shakhova et al*. [2014]. Again, these fluxes were obtained for a different region with different characteristics. On the other hand, when using the more realistic emission area from the CH_4_ hydrate stability model, our maximum possible values are small compared to the other published estimates for the Arctic [*McGuire et al*., 2009; *AMAP*, 2015].

## Supporting information

Supporting Information S1
