# Supplementary material for: Constraints on oceanic methane emissions west of Svalbard from atmospheric in situ measurements and Lagrangian transport modeling
Source: J Geophys Res Atmos. 2016 Dec 10;121(23):14188–200. doi: 10.1002/2016JD025590 (PMC5310218; doi:10.1002/2016JD025590)
Supplement: Supplementary file 1 — Supporting Information S1 [file JGRD-121-14,188-s001.docx]

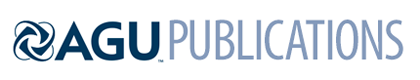


*Journal of Geophysical Research*

Supporting Information for

**Constraints on oceanic methane emissions west of Svalbard from atmospheric in situ measurements and Lagrangian transport modeling**

**I. Pisso^1^, C. Lund Myhre^1^, S. M. Platt^1^, S. Eckhardt^1^, O. Hermansen^1^, N. Schmidbauer^1^, J. Mienert^2^, S. Vadakkepuliyambatta^2^, S. Bauguitte^3^, J. Pitt^4^, G. Allen^4^, K. N. Bower^4^, S. O’Shea^4^, M. W. Gallagher^4, 5^, C. J. Percival^4^, J. Pyle^5, 6^, M. Cain^5, 6^, and A. Stohl^1^**

^1^NILU - Norwegian Institute for Air Research, Instituttveien 18, 2027 Kjeller, Norway

^2^CAGE - Centre for Arctic Gas Hydrate, Environment and Climate, Department of Geology, UiT-The Arctic University of Norway, Dramsveien 201, 9010 Tromsø, Norway

^3^FAAM, Natural Environment Research Council, Building 146, Cranfield, MK43 0AL, UK

^4^School of Earth, Atmospheric and Environmental Sciences, University of Manchester, Manchester, M13 9PL, UK

^5^National Centre for Atmospheric Science (NCAS), UK

^6^Department of Chemistry, University of Cambridge, Lensfield Road, Cambridge CB2 1EW, UK

**Contents of this file**

Text S1

Figure S1

Tables S1 to S6

**Introduction**

This supporting information contains text with details on the instrument drift estimate. The figure shows the potential temperature and suggests that the mixed layer was 1 km high. The tables show flight data analyses (Table 1), the correlations between measured and scenario-based modeled CH_4_ increases (Table 2), up- vs. downwind differences for the transport model analysis (Tables S3 and S4) and flux estimates for the transport model analysis using data form the Zeppelin Observatory in 2014 (Tables S5 and S6)

**Text S1. Estimate of maximum possible instrument drift**

The standard deviation of all target cylinder measurements from FAAM aircraft flights in summer 2014 was 1.41 ppb. Although individual target measurements only comprised of 20 x 1 Hz measurements, this provides a quantification of the drift. The e-folding time of the cell was measured to be 1.4 +/- 0.1 seconds [O'Shea et al., 2013]. Based on this we use 0.33 Hz data to quantify the standard error. In order to estimate the uncertainty, we subtract the standard error of the mean measured CH_4_ mixing ratio from the upwind mean and add the analogous standard error to the downwind mean, on the basis of 3-second mean measurements and assuming that they are statistically independent from each other. Adding this difference to the instrument drift, we find that the true downwind mean can at most be 1.87 ppb (1.6 ppb) higher than the true upwind mean for the first (second) half of the flight. The maximum possible difference between the downwind and the upwind true means is smaller for the second half of the flight because of less variability in the measured CH_4_ and thus a smaller standard error for these data.


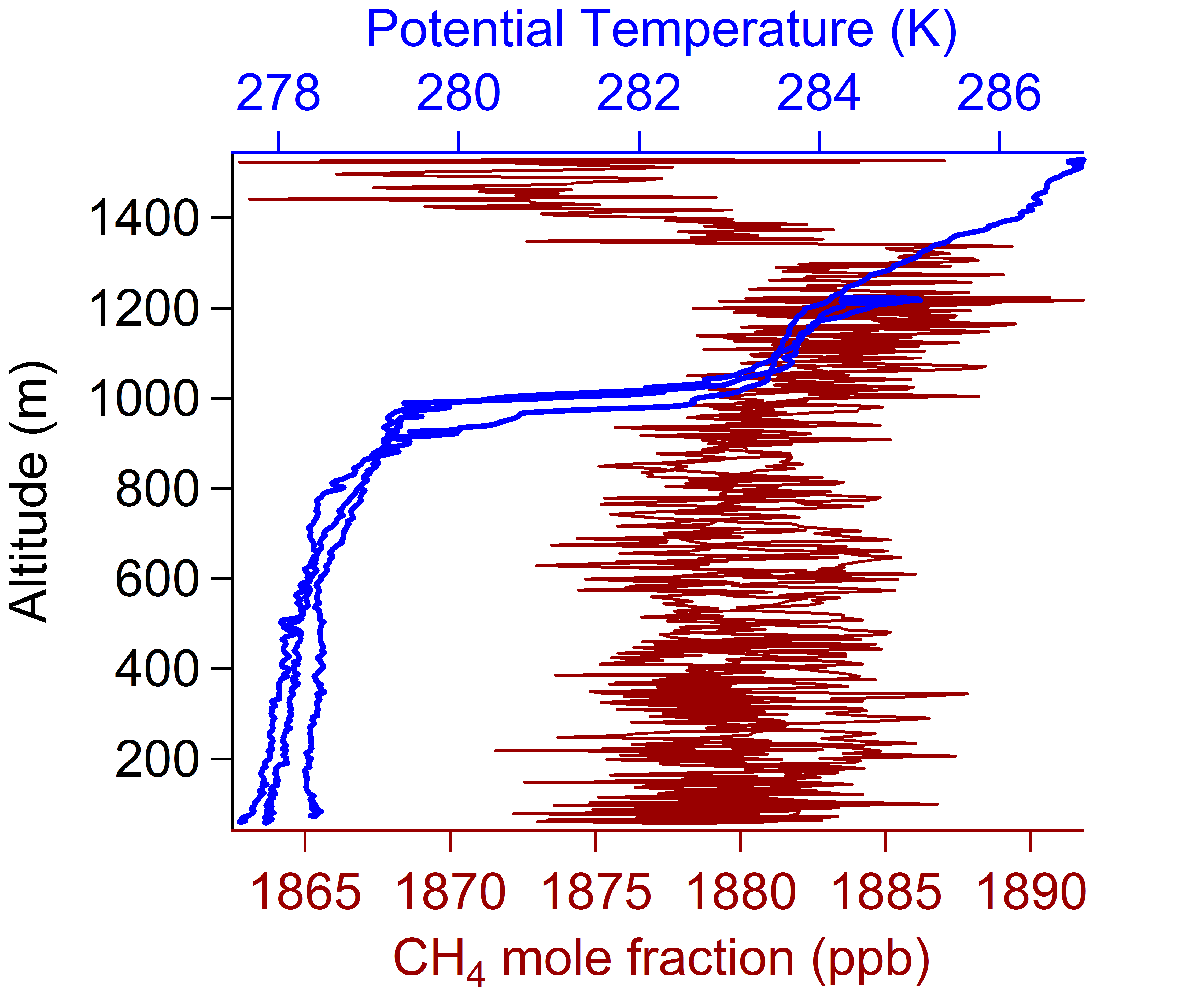


**Figure S1**. Determination of the mixing height during the flight of 2 July 2014 using potential temperature from the aircraft onboard instruments.

Table S1. Mean and standard deviation of CH_4_ mixing ratios measured upwind and downwind of the known seep area during the FAAM flight on 2 July 2014 (see Fig. 5 for definition of upwind and downwind parts of the flight). The null hypothesis for the t-test is that there is no significant difference between the means of the up- and downwind data subsets. For a description of the simulated enhancement from terrestrial sources (ppb), see section 3.2.1.

|  | First half flight | | Second half flight | |
| --- | --- | --- | --- | --- |
|  | Upwind | Downwind | Upwind | Downwind |
| Mean CH_4_ (ppb) | 1879.5 | 1879.3 | 1880.4 | 1880.3 |
| Std CH_4_ (ppb) | 3.16 | 3.63 | 1.79 | 1.63 |
| Sample size | 721 | 600 | 668 | 867 |
| Standard error of the mean for 3 seconds sampling (ppb) | 0.21 | 0.25 | 0.11 | 0.09 |
| *t*-test (unpaired) | No significant difference | | No significant difference | |
| p-value | 0.19 | | 0.057 | |
| Simulated enhancement from terrestrial sources (ppb) | 2.02 | | 1.85 | |

**Table S2.** Correlation coefficients (r) between simulated oceanic mixing ratios and measured (m) mixing ratios, for the three emission region scenarios, during the campaign period (summer 2014). We also provide correlation coefficients for the background-rescaled measurements, with the contribution from terrestrial sources as modeled by FLEXPART subtracted (m_corr_). The rows of the table correspond to different selected data subsets.

| Scenario | Known seeps | | Stability model | | 400 m sea depth | |
| --- | --- | --- | --- | --- | --- | --- |
| Background | m | m_corr_ | m | m_corr_ | m | m_corr_ |
| Aircraft | 0.07 | -0.24 | 0.32 | 0.01 | 0.22 | 0.27 |
| Ship | 0.18 | -0.01 | 0.05 | 0.06 | 0.09 | 0.26 |
| Zeppelin June/July | -0.10 | 0.16 | -0.14 | 0.11 | -0.08 | 0.16 |
| All data | 0.07 | 0 | 0.13 | 0.02 | 0.10 | 0.27 |

**Table S3** Upwind vs. downwind differences for the transport model analysis. Averages and standard deviations (Std) of CH_4_ mixing ratios (ppb) measured when the modeled emission sensitivity in each of the three potential emission regions (see Fig. 4) is in the lowest 20% (“upwind”) and in the highest 20% (“downwind”) of all values. Measurement data from all three measurement platforms are combined.

|  | Known seeps | | Stability model | | 400 m sea depth | |
| --- | --- | --- | --- | --- | --- | --- |
|  | Upwind (lowest 20%) | Downwind (highest 20%) | Upwind (lowest 20%) | Downwind (highest 20%) | Upwind (lowest 20%) | Downwind (highest 20%) |
| Mean CH_4_ | 1878.45 | 1881.33 | 1879.66 | 1880.93 | 1879.68 | 1880.57 |
| Std CH_4_ | 3.73 | 5.31 | 3.80 | 5.47 | 3.67 | 5.34 |
| Sample size | 455 | 455 | 455 | 455 | 455 | 455 |
| Standard error | 0.17 | 0.25 | 0.18 | 0.26 | 0.17 | 0.25 |
| *t*-test (unpaired) | The means are different | | The means are different | | The means are different | |
| p-value | <0.05 | | <0.05 | | <0.05 | |

|  | Known seeps | | Stability model | | 400 m sea depth | |
| --- | --- | --- | --- | --- | --- | --- |
|  | Upwind (lowest 20%) | Downwind (highest 20%) | Upwind (lowest 20%) | Downwind (highest 20%) | Upwind (lowest 20%) | Downwind (highest 20%) |
| Mean CH_4_ | 1872.20 | 1875.66 | 1872.35 | 1875.75 | 1875.28 | 1873.36 |
| Std CH_4_ | 8.92 | 6.30 | 7.44 | 7.79 | 9.08 | 7.64 |
| Sample size | 306 | 306 | 306 | 306 | 306 | 306 |
| Standard error | 0.51 | 0.36 | 0.43 | 0.45 | 0.52 | 0.44 |
| *t*-test (unpaired) | The means are different | | The means are different | | The means are different | |
| p-value | <0.05 | | <0.05 | | <0.05 | |

**Table S4**: Same as Table S3 but with modeled contribution from terrestrial emission sources subtracted from the measurements.

**Table S5**. CH_4_ flux constraints for the three different scenarios and data subsets for Zeppelin in 2014. The average sensitivity is defined as the difference of the sensitivity means (in ppb nmol^-1^m^2^s) for the 20% most and least sensitive points. Modeled contribution from terrestrial emission sources was not subtracted from the measurements.

| Scenario | Known seeps | | | | Stability model | | | | 400 m sea depth | | | |
| --- | --- | --- | --- | --- | --- | --- | --- | --- | --- | --- | --- | --- |
| Estimate (20% most sensitive) | Average sensitivity  (ppb nmol^-1^m^2^s) | CH_4_  increase downwind  (ppb) | Flux constraint | | Average sensitivity  (ppb  nmol^-1^m^2^s) | CH_4_  increase downwind  (ppb) | Flux constraint | | Average sensitivity  (ppb  nmol^-1^m^2^s) | CH_4_  increase downwind  (ppb) | Flux constraint | |
|  |  |  | Flux density (nmol  m^-2^s^-1^) | Total emission  (Gg yr^-1^ ) |  |  | Flux density (nmol  m^-2^s^-1^) | Total emission  Gg yr^-1^ |  |  | Flux density (nmol  m^-2^s^-1^) | Total emission  Gg yr^-1^ |
| Zep. Jan | 0.05 | -6.98 | 127.2 | -106.4 | 0.42 | 5.14 | 12.1 | 177.6 | 0.95 | 6.58 | 6.92 | 804.7 |
| Zep. Apr | 0.02 | 1.27 | 68.45 | 57.24 | 0.19 | 1.46 | 7.71 | 112.9 | 0.45 | -1.1 | -2.45 | -284.3 |
| Zep  Jul | 0.07 | 1.34 | 18.4 | 15.4 | 0.88 | 2.76 | 3.12 | 45.6 | 1.96 | 3.28 | 1.67 | 194.1 |
| Zep Oct | 0.02 | 0.45 | 20.6 | 17.2 | 0.31 | 3.23 | 10.4 | 152.3 | 0.69 | 2.41 | 3.46 | 402.3 |

**Table S6**. CH_4_ flux constraints for the three different scenarios and data subsets for Zeppelin in 2014. The average sensitivity is defined as the difference of the sensitivity means (in ppb nmol^-1^m^2^s) for the 20% most and least sensitive points. Modeled contribution from terrestrial emission sources was subtracted from the measurements.

| Scenario | Known seeps | | | | Stability model | | | | 400 m sea depth | | | |
| --- | --- | --- | --- | --- | --- | --- | --- | --- | --- | --- | --- | --- |
| Estimate (20% most sensitive) | Average sensitivity  (ppb nmol^-1^m^2^s) | CH_4_  increase downwind  (ppb) | Flux constraint | | Average sensitivity  (ppb  nmol^-1^m^2^s) | CH_4_  increase downwind  (ppb) | Flux constraint | | Average sensitivity  (ppb  nmol^-1^m^2^s) | CH_4_  increase downwind  (ppb) | Flux constraint | |
|  |  |  | Flux density (nmol  m^-2^s^-1^) | Total emission  (Gg yr^-1^ ) |  |  | Flux density (nmol  m^-2^s^-1^) | Total emission  Gg yr^-1^ |  |  | Flux density (nmol  m^-2^s^-1^) | Total emission  Gg yr^-1^ |
| Zep. Jan | 0.05 | -10.97 | 199.8 | -167.14 | 0.42 | 9.1 | 21.46 | 314.1 | 0.95 | 7.8 | 8.21 | 953.74 |
| Zep. Apr | 0.02 | 0.44 | 23.86 | 19.95 | 0.19 | 2.16 | 11.4 | 166.9 | 0.45 | 0.96 | 2.13 | 247.6 |
| Zep  Jul | 0.07 | 0.83 | 11.35 | 9.49 | 0.88 | 3.05 | 3.44 | 50.41 | 1.96 | 3.61 | 3.44 | 50.41 |
| Zep Oct | 0.02 | -0.93 | 42.12 | -35.22 | 0.31 | 3.61 | 11.61 | 169.96 | 0.691 | 4.14 | 5.96 | 692.85 |
